# Secreted autotransporter toxin (Sat) induces cell damage during enteroaggregative *Escherichia coli* infection

**DOI:** 10.1371/journal.pone.0228959

**Published:** 2020-02-21

**Authors:** Paulo C. G. Vieira, Abraham O. Espinoza-Culupú, Roberto Nepomuceno, Marina R. Alves, Ivo Lebrun, Waldir P. Elias, Rita C. Ruiz

**Affiliations:** 1 Laboratório de Bacteriologia, Instituto Butantan, São Paulo, SP, Brazil; 2 Laboratório de Bioquímica e Biofísica, Instituto Butantan, São Paulo, SP, Brazil; CINVESTAV-IPN, MEXICO

## Abstract

Secreted autotransporter toxin (Sat) is a 107-kDa serine protease autotransporter of Enterobacteriaceae (SPATE) presenting cytotoxic activity in renal and bladder cells. Further studies have detected the Sat-encoding gene (*sat*) in enteroaggregative *Escherichia coli* (EAEC) and in *E*. *coli* strains isolated from neonatal septicemia and meningitis. Here, we investigated the role of Sat as a cytotoxin of EAEC. Sat was purified from a strain of *E*. *coli* harboring *sat* (DEC/Sat+, O126:H2) and used to raise antibodies in rabbit. The presence of Sat was detected by ELISA in the supernatant of 93.7% of EAEC strains harboring *sat* and in none lacking the gene. The effect of Sat during infection was investigated in polarized Caco-2 cells infected with Sat-producing EAEC (CV323/77, O125ab:H21). This strain induced intense cell detachment, which was inhibited by PMSF or Sat antiserum. Also, *sat* transcription and Sat production were detected during infection. Here we demonstrate that Sat is internalized in polarized cells leading to F-actin disruption which preceded cell detachment. A comparative study of the toxin action in cell lines corresponding to the infection sites in which bacteria carrying the *sat* gene have been isolated was performed. Cells originating from the gastrointestinal tract (Caco-2), urinary (LLC-PK1) and endothelium (HUVEC) were incubated with purified Sat. The time required for observation of cell damage differed according to the cell line. HUVEC cells were more sensitive to Sat than cells derived from urinary and intestinal tracts. The intense activity of Sat on the endothelial cells suggests that Sat could also be a virulence factor for the bacteria in the bloodstream. In addition, this is the first work demonstrating that Sat induces cytotoxic effect during EAEC infection *in vitro*. The cell damage observed during infection indicates that Sat may be another toxin with cytotoxic role in the EAEC pathogenesis.

## Introduction

Whether in the mammalian gastrointestinal tract or any other site of infection, proteases are critical for bacteria to uptake nutritional resources, evade the immune response and colonize the site of infection [1–3]. Members of the serine protease autotransporters of Enterobacteriaceae (SPATE) family are among the group of proteases that have been shown to be relevant in the pathogenicity of different bacteria [4–6].

SPATE comprises a large group of trypsin-like serine proteases secreted by enteropathogens such as *Escherichia coli*, *Shigella*, *Salmonella* and *Citrobacter* species [3,5–7]. These proteins are characterized by the presence of three domains: an N-terminal signal sequence; an extracellular passenger domain, surface exposed or secreted, which exhibits the serine protease GDSGS motif; and a C-terminal β-barrel domain, anchored to the outer membrane [4,8]. These proteins use the type V, or autotransporter (AT), secretion system for exporting to the extracellular space [9]. Phylogenetic analysis clustered SPATE members into two groups: class-1, including those with cytotoxic activities; and class-2, including proteases with mucinolytic and immunomodulatory activities [7].

Sat (secreted autotransporter toxin) is a class-1 SPATE whose passenger domain generates a 107-kDa protein. This protein was first described in an uropathogenic *E*. *coli* (UPEC) isolated from acute pyelonephritis [10]. The role of Sat in urinary tract infection (UTI) was demonstrated in a mice model of ascending UTI with Sat-producing UPEC. Histological changes on glomerular membrane and vacuolation of proximal tubule cells were found [10].

Although Sat’s mechanism of action is not fully understood, the best characterization of Sat to date was obtained in cell lines originated from kidney and bladder. The toxin appears to enter these cells and cleave cytoskeleton-associated proteins [11], where vacuolization and cell elongation were also found [12]. In addition to these, effects related to autophagy induction [13] and degradation of coagulation factor V [14] were described and attributed as an important virulence factor of UPEC.

In diarrheagenic bacteria, most published studies refer only to the detection of *sat* gene. The presence of *sat* has already been described in *Shigella* [10,15], enteropathogenic *E*. *coli* (EPEC) [10,16,17], enterotoxigenic *E*. *coli* (ETEC) [10,16], diffusely adherent *E*. *coli* (DAEC) [13,16,18] and enteroaggregative *E*. *coli* (EAEC) [19,20], in which the protein was identified in culture supernatants by mass spectrometry [19].

Studies correlating Sat toxin with infection of enteric pathogen were performed with DAEC using animal model [21] and polarized intestinal cells [18]. Sat expression by DAEC strain carrying Afa/Dr fimbria induced rearrangement of tight junctions of polarized intestinal cells [18]. Since Afa/Dr DAEC strains are responsible for infection in the gastrointestinal and urinary tract, Sat could be an important virulence factor in both infection niches [18]. Also, purified Sat from culture supernatant of a probiotic *E*. *coli* (Nissle 1917) altered the permeability of polarized Caco-2 cells [22]. On the order hand, infection of polarized Caco-2 cells by the Nissle 1917 strain did not affect cell permeability, suggesting that Sat does not act as a virulence factor in the intestine when present in commensal *E*. *coli* [22]. These findings clearly demonstrated that the action of native Sat may be dependent of the bacterial background [22].

More recently, different studies have found *sat* in bacterial strains originated from sites of infection other than the urinary tract and intestine [23–29]. Similarly, *sat* has been described as the second most prevalent SPATE gene in *E*. *coli* strains isolated from neonatal sepsis [23] and detected in an *E*. *coli* strain causing a fatal neonatal meningitis [24]. These findings suggest that Sat may have some relevance in the pathogenesis of these bacteria and show the relevance of expanding the study of cytotoxicity by comparing cell lines originated from possible niches of infection.

In this study we investigated: i) the expression of Sat by EAEC strains isolated from diarrhea; ii) the cytotoxic effect of Sat during infection *in vitro* by EAEC iii) Sat internalization in polarized Caco-2 intestinal cells iv) the kinetics of cell damage induction by purified Sat in cell lines derived from tissues representing infection niches where *sat*+ bacteria have been isolated.

We demonstrated for the first time that Sat is secreted during EAEC infection and induces cell detachment in polarized intestinal cells. In addition, purified native Sat induced cell detachment differing according to the origin of the cell line, *i*.*e*. endothelial cells being more sensitive than cells originated from the urinary and intestinal tract.

## Materials and methods

### Bacterial strains and growth conditions

For this study we used the following *E*. *coli* strains: EC233/93, serotype O126:H2 (DEC/Sat+) [30] and CV323/77, serotype O125ab:H21 (EAEC) [31], both isolated from cases of acute diarrhea in children in São Paulo; and a collection of 20 EAEC strains isolated in an epidemiological study of the etiology of acute diarrhea in children conducted in Bahia, Brazil [20]. Among these 20 strains, 16 were positive for *sat* detection and 4 were negative [20]. The strains DAEC FBC114 [16], EAEC prototype strain 042 [32], *E*. *coli* HB101(pDG7) (*sat* cloned into pBSKII) [10] and *E*. *coli* HB101 (non-pathogenic) [33] were used as controls.

Bacteria were grown in Luria-Bertani (LB) (Gibco, Rockville, MD) agar or LB broth before each experiment. For the cytotoxicity assays, 100 𝜇L bacterial cultures were seeded in 3 mL Dulbecco’s modified Eagle’s medium (DMEM) or DMEM containing 1% tryptone (DMEM-tryptone) and grown at 37°C with shaking at 200 rpm, until the exponential phase. The HB101/Sat strain was maintained in the presence of ampicillin.

### Cell cultures

Y1 cells (ATCC CCL-79), LLC-PK1 cells (ATCC CL-101) and HEp-2 cells (ATCC CCL-23) were grown in DMEM) high glucose (Cultilab, Campinas, São Paulo) supplemented with 10% fetal bovine serum (FBS) (Cultilab, Campinas, São Paulo). Caco-2 cells (ATCC HTB-37) were grown in DMEM supplemented with 15% FBS and HUVEC cells (ATCC CRL-1730) were grown in RPMI (Cultilab Campinas, São Paulo) supplemented with 10% FBS plus endothelial cell growth supplement from bovine neural tissue (ECGS) (Sigma, St. Louis, Missouri, USA). These cells were maintained in humidified atmosphere with 5% CO_2_, at 37 ºC. The subcultures were harvested with trypsin-EDTA (Gibco) pH 7.4, washed and distributed at 2 x 10^5^ cells/well in 24-well culture plates (Corning Incorporated Costar®—USA) containing 13 mm round cover glasses or 5 x 10^4^ cells/well in 96-well culture plates (Corning Incorporated Costar®—USA). For polarization of Caco-2 cells, the cultures were maintained in 24-well plates for 15 days. The culture medium was changed every 48 h, when cells were confluent they were washed twice with phosphate buffered saline (PBS) [34]). HEp-2 cells were used in adhesion assays and the other lines were used in the cytotoxicity assays.

### HEp-2 cells adherence test

The adherence pattern of the bacteria on HEp-2 cells was determined following the adhesion assay described by Cravioto et al. [35]. Briefly, HEp-2 cells cultivated for 48 h (80% confluence) in DMEM supplemented with 2% fetal bovine serum and 1% D-mannose were infected with bacteria grown overnight in LB broth (MOI 100). After 3 h-incubation at 37°C, the infected cells were washed and again incubated for a further 3 h, with fresh DMEM supplemented with 2% fetal bovine serum and 1% D-mannose. Cultures were then washed with PBS, fixed with methanol, stained with May-Grümwald and Giemsa and examined by light microscopy (1,000X).

### Detection of virulence genes

Bacterial DNA samples were obtained from overnight cultures on LB agar incubated at 37°C, from which single colonies were resuspended in 300 μL of MilliQ water and boiled for 10 min. The PCR was used to detect the presence of characteristic markers of EPEC (*bfpA*, *eae* and *perABC*) [20,36]; EAEC (*aggR* and *aatA*) [20,37]; members of the SPATE family (*eatA*, *espC*, *epeA*, *espI*, *espP*, *pet*, *pic*, *sat*, *sepA*, *sigA*, *tsh* and *vat*) [17,20,38]; and of other autotransporters (*aida-I*, *cah*, *ehaA*, *ehaB*, *ehaC*, *ehaD*, *ehaJ*, *sab* and *tibA*) [17,20,38]. The primers used were described in the respective references.

Amplification was performed in a total volume of 25 μL containing: 20 pmol of each primer; 1.0 U Taq DNA polymerase; 5.0 μL 10X PCR buffer (200 mM MgCl_2_ [pH 8.4], 500 mM KCl); 1.0 μL of DNA template, employing the controls and amplification cycles previously described [17,20,36,38,39].

### DNA sequencing and analysis

The 930 bp amplified fragments corresponding to *sat* of EAEC CV323/77 and DEC/Sat^+^ strains were sequenced in a Mega-BACE 1000 (Amersham Pharmacia Biotech) sequencer. Reactions were performed according to the manufacturer’s instructions, using the APBiotech DYEnamic ET Dye Terminator Cycle Sequencing Kit. Sequence analysis was performed using the MEGA Version 7.0.26 software (Analysis Expert DNA Star Software, Inc. PC, Madson, WI, USA) and BioEdit Sequence Alignment Editor 7.0.5.3 (Carlsbad). The identity of the nucleotide sequence of the 930 bp fragment of both strains with CFT073 (GenBank accession number AF289092.1) was searched using the BLASTn program.

### Purification of Sat

Overnight of LB broth (1,000 mL) of DEC/Sat+ strain, grown until the optical density (OD) reached 1.0 at 600 nm, was used to obtain purified Sat. Bacteria were removed by centrifugation at 10,000 x *g* for 10 min at 4°C. Supernatants were sterilized through a 0.22 μm pore-sized filter. Samples were concentrated using tangential filtration flow (Labscale TFF system) (50,000 molecular weight cut-off) to a volume of approximately 50 mL. The supernatant was again concentrated using vivaspin 20 (50,000 molecular weight cut-off) (GE healthcare) to a volume of 10 mL. The concentrated was loaded in the serine protease affinity column (HiTrap^TM^ Benzamidine FF High sub, GE Healthcare, Sweden, catalog number 17-5144-01), previously equilibrated with binding buffer (0.05 M Tris-HCL, 0.5 M NaCl, pH 7.4). The column was washed with 25–50 mL of binding buffer or until no material appeared in the eluent (monitored by UV absorption at A_280 nm_ on the AKTA Prime Plus, GE). Protein was eluted with 25–50 mL of elution buffer (0.05 M glycine-HCl, pH 3.0) at a flow rate of 4 mL/min. Fractions of 1 mL were collected in presence of 20 μL of neutralization buffer (1 M Tris-HCl pH 9.0). A single band of 107 kDa was visualized by sodium dodecyl sulfate-polyacrylamide gel electrophoresis (SDS-PAGE). The fractions containing the protein consistent with the predicted molecular mass of Sat (107 kDa) was pooled and concentrated by ultrafiltration Vivaspin20 (GE Healthcare). Protein concentration was determined using a bicinchoninic acid assay (Pierce, Rockford, Illinois). Mass spectrometry (LC-MS/MS) confirmed Sat identity.

### Production of polyclonal antibody against Sat

New Zealand rabbit (60-day-old) was supplied by the Animal Research Facilities of the Butantan Institute. Rabbit was immunized with three doses of 100 μg of purified Sat protein containing 2.5 mg of aluminum hydroxide as adjuvant. Blood was collected at the 45^th^ day, and the presence of antibodies was confirmed by Western blotting. All procedures involving the use of animal were performed according to the Care and Use of Laboratory Animal Guidelines (1996) and were approved by the Ethics Committee on Animal Use of the Butantan Institute (CEUAIB Certificate 1362/15).

### Bacterial infection

Cultures of Y1 and polarized Caco-2 cells were infected in the absence of FBS, at a MOI of 10 with the EAEC CV323/77, DAEC FBC114, *E*. *coli* HB101(pDG7) and *E*. *coli* HB101 bacterial strains grown in DMEM-tryptone for approximately 3 h. After 5 h of infection, the cells were used for cytotoxicity evaluation, while the bacteria were used for analysis of transcription of *sat* by RT-PCR and the culture supernatant used to investigate the expression of Sat by Western Blotting. The culture supernatant of strain EAEC 042 was used in Western Blotting to evaluate whether anti-Sat cross-react with Pet or Pic, and purified Sat was used as positive control.

### RNA extraction and RT-PCR amplification

The supernatant of the infected Y1 cells were centrifuged to obtain the bacteria. Total RNAs from bacteria were extracted and purified using QiAzol Lysis Reagent (Qiagem ®) and Direct-Zol^™^ RNA MiniPrep Plus (Zymo Research), according to the manufacturer´s instructions. RNAs were quantified using GE NanoVue Plus^TM^ Spectrophotometer (GE Lifesciences) and the quality control of the extracted RNAs was assessed by agarose gel electrophoresis post stained with gelRed ^TM^ (Biotium). Total RNAs (200 ng) were converted to cDNA using High Capacity RNA-to-cDNA Kit according to the manufacturer's instructions (Applied Biosystems^TM^). cDNAs (10 ng) were used as template for amplification of *sat* and *icd* (as internal control) by PCR using the following primers: *sat* (F) 5'-TCAGAAGCTCAGCGAATCATTG-3' and *sat* (R) 5'-CCATTATCACCAGTAAAACGCACC-3', to amplify a 930 bp fragment [19]; and *icdA* (F) 5'-CTGCGCCAGGAACTGGATCT-3' and *icdA* (R) 5'-ACCGTGGGTGGCTTCAAACA- 3', to amplify a 669 bp fragment [40]. For amplification, 5 μL of cDNA were used as template in reactions as described above for the detection of virulence genes. The RT-PCR products were analyzed by agarose gel (1.2%) electrophoresis post stained with GelRed ^TM^. Images of the RT-PCR agarose gels were acquired using an electronic documentation system (UVITEC, Cambridge).

### Detection of Sat expression during infection

In order to confirm the expression of Sat during infection, the polarized Caco-2 cells culture media was removed, centrifuged at 5,500 x *g* and precipitated with trichloroacetic acid (TCA). The precipitates were then submitted to SDS-PAGE and electrotransferred onto a nitrocellulose membrane (Millipore). Membrane was then incubated with primary rabbit anti-Sat serum (1:500 dilution), followed by incubation with peroxidase-conjugated anti-rabbit IgG (1:5,000 dilution). Positive signals were detected by enhanced chemiluminescence (West Pico, Pierce).

### Detection of Sat production by indirect enzyme-linked immunosorbent assay (ELISA)

Each one of the 20 strains from the EAEC collection (16 *sat*+ and 4 *sat*- strains) were grown in 3 mL of LB broth at 37°C overnight, with constant shaking (200 rpm). In the following day, 100 μL of bacterial cultures adjusted for OD 300 were inoculated in 3 mL of medium and grown in the same conditions.

The supernatants of the cultures were obtained after centrifugation at 15,700 × *g* for 10 min, and used for coating ELISA microplates (Corning Incorporated Costar®, high binding) (USA). After coating (4°C for 18 h), the wells were blocked with 5% nonfat milk in PBS, and incubated with anti-Sat rabbit serum (1:100) followed by peroxidase conjugated goat anti-rabbit IgG antibody (1:5,000) incubation. The assay was developed with o-phenylediamine (OPD) and H_2_O_2_, and the absorbance was measured at 492 nm in a Multiskan EX ELISA reader (Labsystems, USA). The strains DEC/Sat+, EAEC CV323/77 and DAEC FBC114 were used as positive control and strain HB101 as negative control. All strains were tested in triplicates and at least two independent experiments were performed.

### Kinetics of the cytotoxic effect of purified Sat

Y1 cell cultures maintained in 24-well plates were washed twice with PBS and then incubated with purified Sat at the concentration of 50 or 100 μg/mL for 2, 5, 8 or 24 h at 5% CO_2_-95% air at 37°C. After the different times of infection, the cultures were washed with PBS, fixed with methanol, stained with May-Grümwald and Giemsa and used in the evaluation of cell morphology and detachment by light microscopy (400X).

### Cytotoxicity assays

Cytotoxicity assays were evaluated in both infected cells and incubated with the purified Sat. Infection was performed on Y1 and polarized Caco-2 cells and assays with purified Sat were performed on Y1, Caco-2, LLC-PK1 and HUVEC lines cell incubated with 100 μg /mL toxin at different incubation times.

After incubation, the culture medium was aspirated and the cells were washed twice with PBS. Both for the evaluation of the morphologic effect and for the determination of the percentage of the adhered cells, the cultures were fixed with 70% methanol, stained with 0.1% Giemsa (Sigma-Aldrich, St. Louis, MO) and evaluated by light microscopy.

The morphologic effect was determined based on the criteria described in previous works [41,42], where a score of 1+ indicated the presence of elongated or rounded cells greater than those observed in the control (but with less than 50% of cells affected); 2+ indicates that 50% of the cells are rounded but detachment was less than 50%; 3+ indicates that more than 50% of the cells were detached and all remaining cells were rounded; and 4+ indicates that all (or nearly all) cells were detached from the glass.

To determine the percentage of adhered cells after infection or toxin treatment, we quantified cells of 6 random fields (400X). The number of cells obtained in the negative controls was considered 100% adherence. Cells infected with *E*. *coli* HB101 or that did not receive the toxin served as negative controls. All experiments were performed at least three times, each time in triplicate.

#### Cell viability assay

Both membrane permeability and mitochondrial metabolism were assessed by Trypan blue exclusion and MTT methods, respectively. The viability of detached cells after incubation with the purified Sat (100 μg/mL) was determined by the Trypan blue exclusion method. The Trypan blue exclusion dye (Sigma-Aldrich) that is only incorporated by cells with altered permeability was added (1:2) in the cell cultures for 20 min at 37 ^o^C. The percentage of viable, non-stained cells was determined after quantifying 300 cells by light microscopy. On the other hand the viability of cells that remained adhered after incubation with the toxin was assessed by MTT and Trypan blue exclusion method. The MTT assay, which is based on the metabolic reduction of 3-(4,5-dimethylthiazol-2-yl)-5-(3-carboxymethoxyphenyl)-2-(4-sulfophenyl)-2H-tetrazolium by mitochondrial enzyme activity of viable cells, was determined according to the manufacturer’s instructions (Sigma-Aldrich). The results are representative of at least three independent experiments, each one in triplicate.

#### Neutralization of the cytotoxic effects

Cytotoxic effect neutralization assays were performed on polarized Caco-2 and Y1 cells cultures incubated with bacteria or purified Sat. The neutralization test was performed using anti-Sat IgG or the serine protease inhibitor phenylmethylsulfonyl fluoride (PMSF) (Boehringer, Indianapolis, IN).

The strains grown in DMEM-tryptone, at 37°C (OD_600nm_ = 0.3) were preincubated with 250 μg/mL anti-Sat. Alternatively, the same cultures were preincubated with 0.6 mM PMSF. The positive controls of infection were DAEC FBC114 or *E*. *coli* HB101(pDG7) and *E*. *coli* HB101 was used as negative control. The purified toxin (100 μg/mL) was preincubated with anti-Sat at the concentration of 75 μg/mL or PMSF at the concentrations of 0.3 and 0.6 mM. In both cases the preincubation times were 30 min with the antibody and 15 min with PMSF. All preincubated aliquots were then added to the cells in fresh medium and incubated for 5 h (infection) or 8 h (purified-Sat) at 37 ºC prior to standard fixation and staining. Positive controls were cells infected or incubated with toxin in the absence of PMSF or anti-Sat and negative controls were cells only or cells incubated with PMSF or anti-Sat. All experiments were performed at least three times, each time in triplicate.

### Actin filament analysis

Polarized Caco-2 cells were incubated with 100 μg/mL of purified Sat. After incubation for 6 h, the cells were washed with PBS-0.005% Tween-20. Then, the cells were incubated for 30 min, at room temperature, with a solution containing 4% paraformaldehyde (PFA), 0.3% Triton-X100, 1% BSA and 0.005% Tween 20. Actin filaments were stained by rhodamine phalloidin (1:40) (Thermo Fisher Scientific) and nucleus stained by 4´-6-diamidino-2-phenylindole dihydrochlotide (DAPI) (1:300) (Sigma-Aldrich). Actin filaments were analyzed by Leica DMi8 inverted fluorescence microscopy, coupled to DFC310 FX digital camera (Leica Application Suite Software—LAS V4.6).

### Toxin Labeling and confocal laser-scanning microscopy

Two hundred μg of purified Sat toxin (1 mL) were added in 4 mL of HEPES solution (10 mM pH 8.3) containing 300 μg FITC (Sigma-Aldrich). The pH of the reaction buffer was adjusted to 8.3 by using sodium bicarbonate. The reaction was carried out on ice in the dark for 3 h under continuous stirring. After this period, the reaction was stopped by the addition of 5 mL of NH_4_Cl solution (50 mM). The labeled toxin was concentrated by Vivaspin 20 (50,000 molecular weight cut-off) (GE healthcare) and the free FITC was removed by three consecutive washes with HEPES solution. The internalization of the Sat was visualized by confocal fluorescence microscopy (Leica TCS SP8) and images were obtained via LASX computer software.

### Statistical analysis

All data were analyzed using Graph- Pad Prism 6.0 (San Diego, CA). The 2-way ANOVA with Tukey’s multiple comparison test was carried out. Results were considered statistically significant when 𝑃 < 0.05.

## Results

### Characterization of bacterial strains

**Fig 1:**
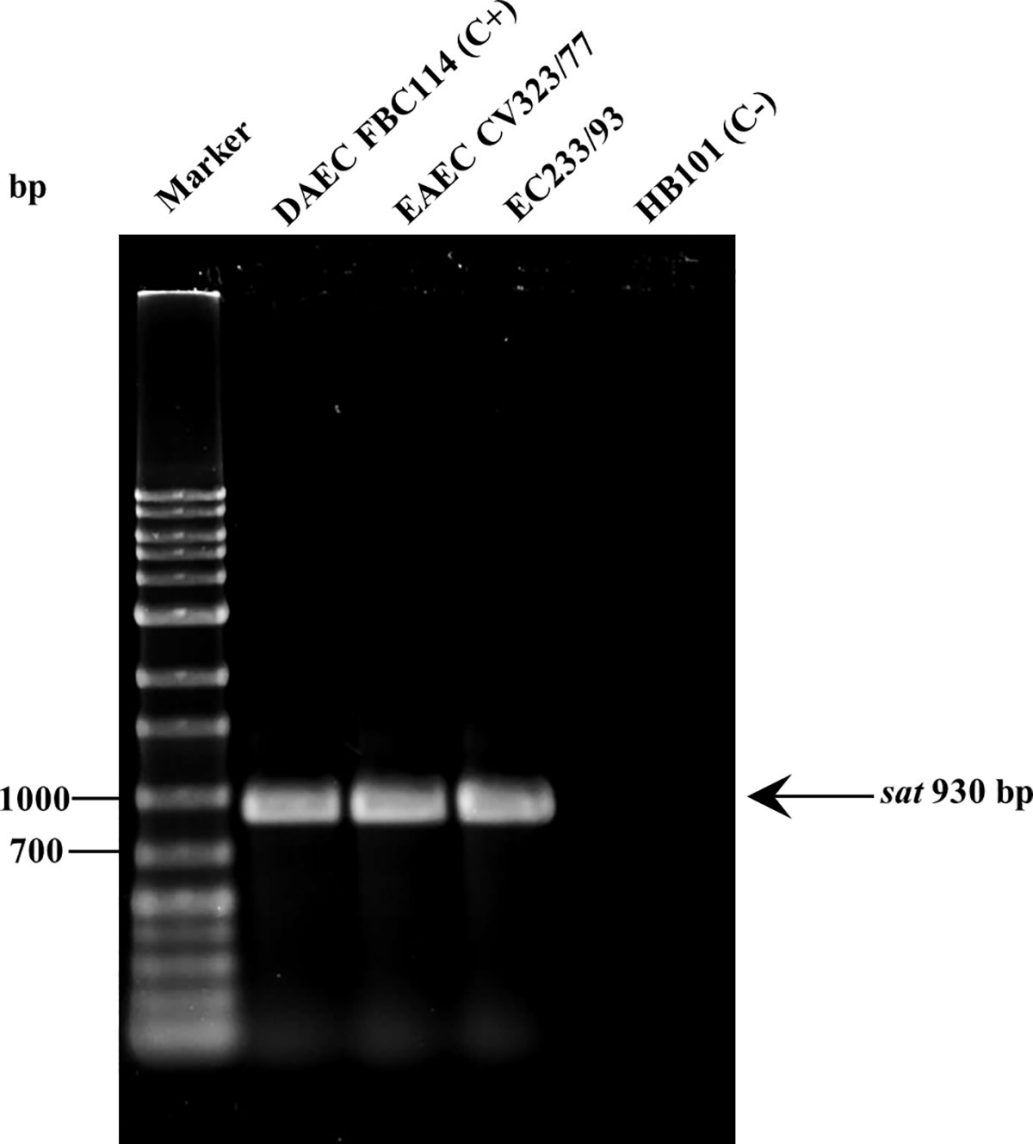
Amplification of *sat* in *E*. *coli* strains by PCR. DAEC FBC114 –positive control, HB101 –negative control, CV323/77 and EC233/93—tested strains. Marker: 1 kb Ladder Plus (Sinapse Inc).

In the present study, we amplified a 930 bp fragment corresponding to *sat* gene in two strains of *E*. *coli* isolated from cases of childhood diarrhea: EC233/93 (serotype O126:H2) and CV323/77 (serotype O125ab:H21) strains (Fig 1).

To better characterize these two strains, we investigated the characteristic virulence markers of EAEC (*aatA*, *aggR*), EPEC (*bfpA*, *perABC* and *eae*) and genes of autotransporter proteins of *E*. *coli* (*espP*, *pet*, *espC*, *sigA*, *eat*, *vat*, *epeA*, *espI*, *pic*, *tsh*, *aida-I*, *cah*, *ehaA*, *ehaB*, *ehaC*, *ehaD*, *ehaJ*, *sab* and *tibA*). In both strains, *sat* was the only amplified gene that corresponds to a cytotoxic protein (Table 1). Amplification of *aatA* in the absence of *aggR* classified the CV323/77 strain as an atypical EAEC (Table 1). The confirmation of the pathotype was also obtained by observing the aggregative adhesion (AA) pattern on HEp-2 cells, characteristic of EAEC (Table 1). On the other hand, in EC233/93 strain, which was non-adherent on HEp-2 cells (Table 1), none of the virulence markers were amplified, except *sat* (Table 1). The absence of virulence markers from other diarrheagenic *E*. *coli* pathotypes had already been shown in both strains [30,31]. Thus, the CV323/77 and EC233/93 strains were named as EAEC CV323/77 and DEC/Sat^+^, respectively.

**Table 1:** Characterization of EAEC CV323/77 and DEC/Sat+ strains.

	Markers	Strains		Function/effect	Reference
		O125ab:H21	O126:H2		
		EAEC CV323/77	DEC/Sat+ EC323/93		
**EAEC**	*aatA*	+	-	Dispersin transporter	[46]
	*aggR*	-	-	Transcriptional activator	[38]
	Adhesion pattern	AA*	NA**		[32]
AT^***^	*sat*	**+**	**+**	Cytotoxic	[10]
	*espI*	+	-	Mucinase	[37]
	*pic*	+	-	Mucinase	[57]
	*cah*	+	-	Biofilm formation	[58]
	*eha A*	+	-	Biofilm formation	[59]
	*eha J*	+	-	Biofilm formation	[60]

* Aggregative Adherence pattern

** Non-Adherent

*** Autotransporter

The 930-bp amplicon from the two strains were sequenced and aligned using the MEGA 7 program. The entire *sat* gene corresponds to 3,900 bp in UPEC prototype strain CFT073 (GenBank accession number: AF289092.1) and the 930-bp amplicon alignment occurred between positions 1,771 to 2,613 of the *sat* sequence of strain CFT073. We identified four mutations in both amplicon sequences, but only two of them resulted in amino acid exchange (Table 2). In the *in silico* analysis, both the hydrophobicity [43] and the recognition factors [44] presented modifications when compared with the reference strain. The *sat* amplicon sequences were submitted to GenBank under the accession numbers LT855558 (EAEC CV323/77) and LT855559 (DEC/Sat^+^).

**Table 2:** Amino acid residues from Sat which differ from reference strain CFT073.

Origin	Strain	Serotype	Sat amino acid residue at position		Reference
			669	771	
**Blood/ Urine**	CFT073	O6:K2:H1	V	T	[10]
**Probiotic**	EcN	O6:K5:H1	A	T	[22]
**Diarrhea**	DEC/Sat+	O126:H2	A	A	This study
**Diarrhea**	EAEC CV323/77	O125ab:H21	A	A	This study

### Identification and purification of Sat

Concentrated bacterial culture supernatant of the DEC/Sat+ strain was submitted to SDS-PAGE analysis. The band corresponding to ~107 kDa molecular mass was sequenced by mass spectrometry LC-ESI-MS (MS/MS). The interpretation of the mass spectra for the deduction of the peptide sequences and subsequent identification of the proteins were performed using Mascot software, version 2.4.1. The peptide sequences were compared to the corresponding sequence of UPEC CFT073 (GenBank accession number: AF289092.1). The portion of the sequenced protein corresponds to the translocated domain (amino acid position from 50 to 1,018). The analysis score was 1,934, confirming that the analyzed peptides corresponded to the Sat protein.

**Fig 2:**
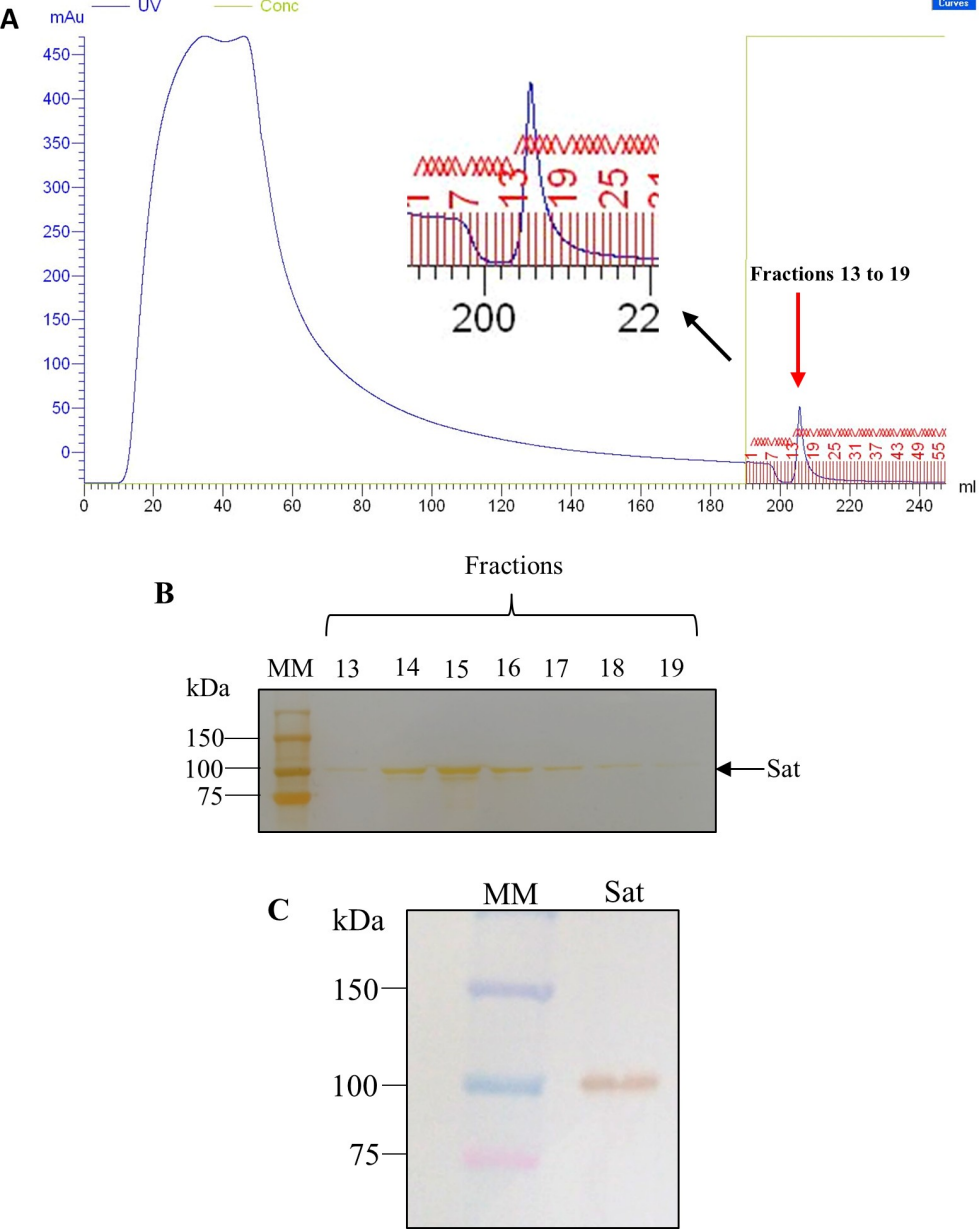
Purification of Sat. A) Chromatogram of strain DEC/Sat+ supernatant using serine-protease column HiTrap^TM^ Benzamidine FF High sub (GE Healthcare). As indicated in the figure, the fractions 13 to 19, corresponding to the highlighted peak, were collected. B) SDS-PAGE of the fractions collected after purification indicating the presence of Sat protein (Silver staining). C) Western blotting of the fractions pooled and concentrated by ultrafiltration Vivaspin20 (GE Healthcare). Precision Plus Protein^TM^ Standard (BioRad).

Concentrated bacterial culture supernatant was submitted to the serine protease affinity column. In the fractions collected from 13 to 19, which corresponded to peak in the chromatogram (Fig 2A), the ~107 kDa band was visualized (Fig 2B) and detected by Western blotting using the anti-Sat serum (Fig 2C).

### Cytotoxic action of Sat during the infection by EAEC

**Fig 3:**
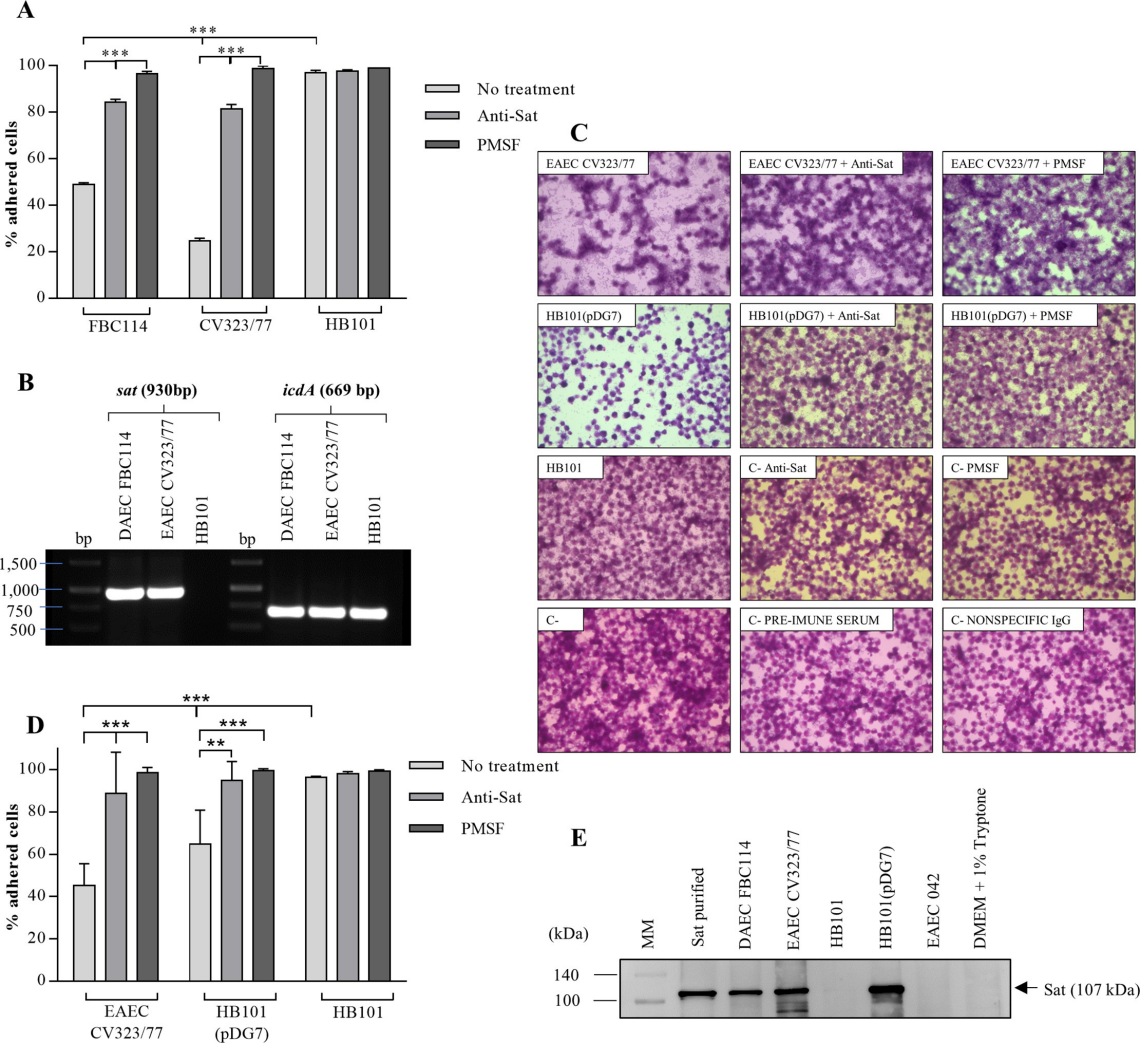
Evaluation of Sat induced cell damage after 5 h of infection with EAEC CV323/77. A) Percentage of adhered Y1 cells after infection B) Reverse transcriptase chain reaction for *sat* during infection of Y1 cells. Isocitrate dehydrogenase gene (*icdA*) was used as an internal control. C) Observation of cell damage in cultures from the polarized Caco-2 cells by light microscopy. Magnifications 400X. D) Adhered cell percentage after infection of polarized Caco-2 cells in the presence or absence of PMSF (0.6 mM) or polyclonal anti-Sat antibody (250 μg/mL). E) Immunodetection of Sat in the supernatant collected from polarized Caco-2 cells by Western Blotting. Strains used as controls were *E*. *coli* HB101(pDG7) (positive control) and *E*. *coli* HB101 (negative control). Statistical analysis was performed by ANOVA (***p<0.001) with GraphPad Prism program.

To investigate whether infection with the EAEC CV323/77 strain induced cellular damage due to Sat secretion, we initially infected the Y1 (adrenal-derived) cell line, described as Sat sensitive [21]. Cells were infected (MOI 10) for 5 h in the presence or absence of anti-Sat IgG or PMSF, a serine protease inhibitor. Cell detachment was analyzed by light microscopy and the percentage of adherent cells after infection was determined (Fig 3A). Cells infected with DAEC FBC114 were used as positive control. Cell detachment was observed in cultures infected with EAEC CV323/77 and FBC114 strains in the absence of anti-Sat or PMSF (Fig 3A). Cell detachment was significantly inhibited when the cells were infected with the strains incubated with anti-Sat or PMSF (Fig 3A). Transcription of *sat* was demonstrated in the bacteria recovered post-infection from the culture medium, using semi-quantitative RT-PCR (Fig 3B). Considering that EAEC is a diarrheagenic pathotype we also evaluated the action of EAEC CV323/77 strain on polarized Caco-2 intestinal cells, after 5 h of infection. As control of Sat effects, cells were also infected with the *E*. *coli* HB101(pDG7), the *sat* minimal clone. Both EAEC CV323/77 and *E*. *coli* HB101(pDG7) strains induced a significant cellular detachment of Caco-2 cells (Fig 3C). On the other hand, infection in the presence of PMSF or anti-Sat, no cellular detachment was observed (Fig 3C and 3D). Sat toxin was detected in the supernatant of infected Caco-2 cell cultures (Fig 3E) concomitantly with detection of morphological changes (Fig 3C) and cell detachment (Fig 3D). Cells incubated with the same culture medium (with or without PMSF and anti-Sat), without infection, or infected with *E*. *coli* HB101 were used as negative controls and considered as 100% of adhesion. Neither anti-Sat IgG nor PMSF induced any cell detachment (Fig 3C). As shown in Fig 3E, the anti-Sat serum does not cross-recognize either the Pet or Pic toxin. Both proteins are autotransporters which exhibit Sat homology and are secreted by the EAEC 042 prototype strain. Neither the pre-immune serum nor nonspecific IgG fractions induced any cell detachment (Fig 3C).

### Secretion of Sat by different strains of EAEC

**Fig 4:**
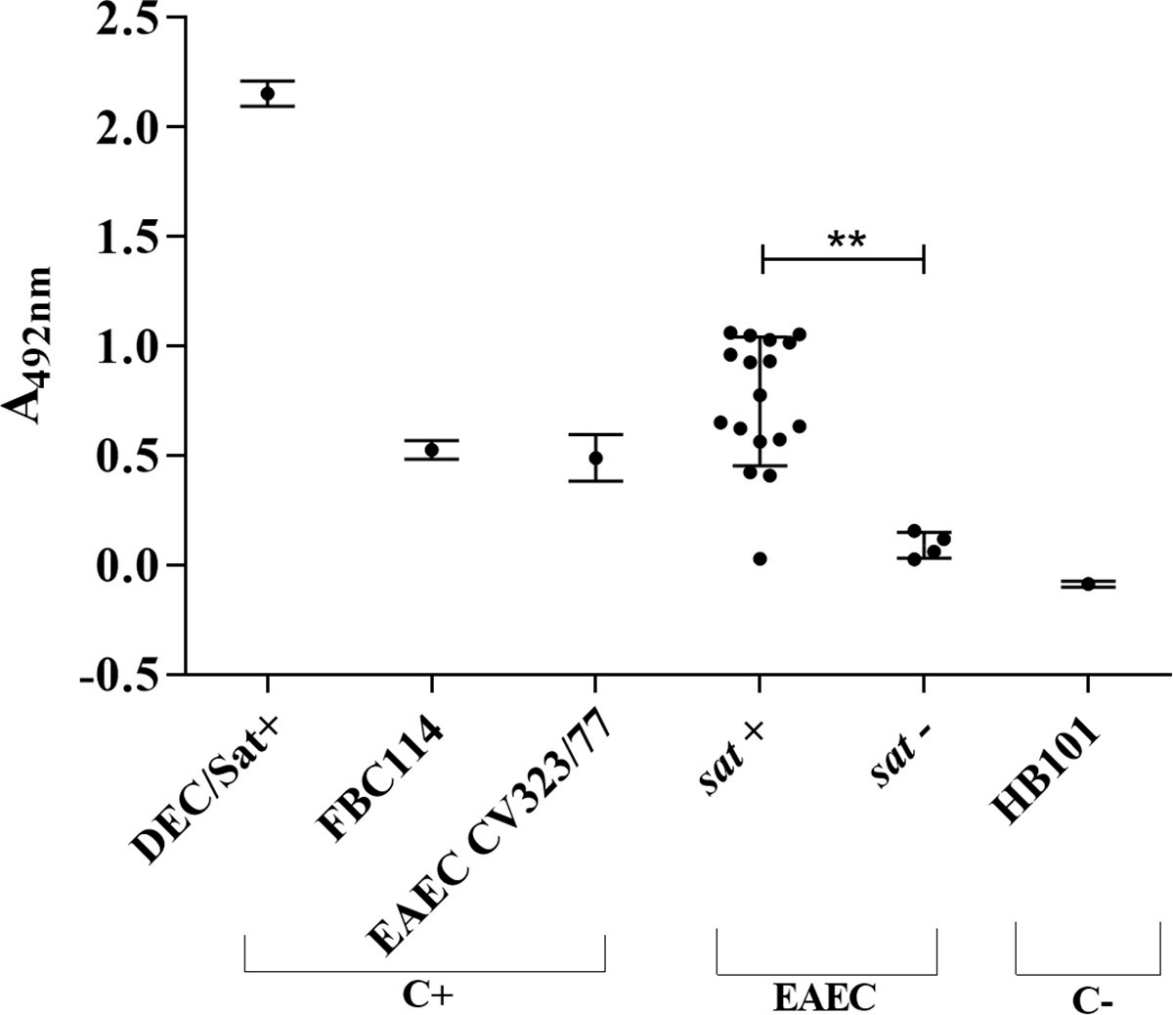
Investigation of Sat secretion in a collection of EAEC strains. Culture supernatants of 20 bacterial strains (16 *sat+* and 4 *sat*- strains), grown in DMEM, were tested by indirect ELISA for Sat detection using anti-Sat rabbit serum. The strains DEC/Sat+, EAEC CV323/77 and DAEC FBC114 were used as positive controls, and *E*. *coli* HB101 as negative control. All strains were tested in triplicates and at least two independent experiments were performed. The data shown are the absorbance values (mean ± standard error) for each strain from a representative experiment.

In order to verify if the secretion of Sat by EAEC was not exclusive to the EAEC CV323/77 strain, the production of Sat was also investigated by ELISA in culture supernatants from a collection of 20 EAEC strains (16 *sat*+ and 4 *sat*- strains) isolated from children with diarrhea [20]. The strains DEC/Sat+, EAEC CV323/77 and DAEC FBC114 were used as positive control and four *sat*-negative EAEC were used as negative controls [20]. The supernatant of the bacterial cultures were tested by ELISA using anti-Sat anti-serum. Sat was detected in the supernatant of 15 (93.7%) *sat*-positive EAEC and in none of the *sat*-negative EAEC (Fig 4).

### Cytotoxic activity of purified toxin

**Fig 5:**
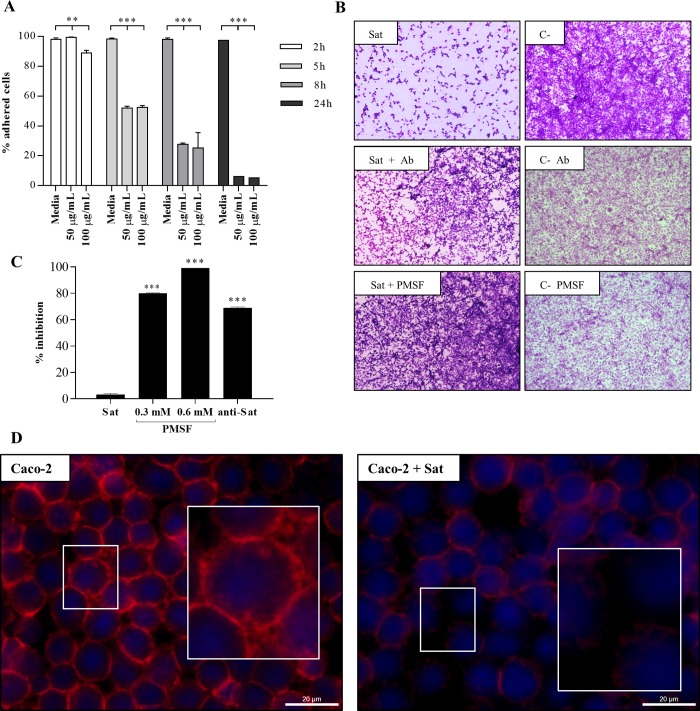
Effect of purified Sat on cells. A) Kinetics of cytotoxicity in Y1 cells incubated with 50 or 100 μg/mL of Sat at 2, 5, 8 and 24 h. B) Cultures incubated for 8 h with 100 μg/mL of Sat previously treated with PMSF (0.6 mM) or anti-Sat polyclonal antibody (75 μg/mL). The positive control was cells incubated with non-treated Sat and negative control was cells incubated with DMEM plus elution-buffer. Observation of inhibition of cell damage by light microscopy. Magnifications 100X. C) Percentage of Y1 cell detachment inhibition after incubation with Sat previously treated with PMSF (0.3 and 0.6 mM) or polyclonal anti-Sat antibody (Ab). D) Actin filaments of polarized Caco-2 cells incubated for 6 h with Sat 100 μg/mL were labeled by phalloidin rhodamine (red). Cell nuclei were stained by DAPI (blue). The large squares correspond to the same areas of the small squares, but with higher magnification. Observation by fluorescence microscopy. Magnifications 1,000X. Bar: 20 μm. Statistical analysis was performed by ANOVA (***p<0.001) with GraphPad Prism program.

To investigate the activity of purified Sat we performed kinetics of action on Y1 cell line considering its sensitivity to Sat showed in previous work [21]. Cultures were incubated with 50 or 100 μg/mL of Sat at 2, 5, 8 and 24 h. Cell damage was analyzed by light microscopy. Sat induced cell detachment in a time-dependent manner, after 24 h of incubation, and the detachment was nearly 100% (Fig 5A). Cell detachment was similar in the cultures incubated with both concentration of the toxin, except at the point of 2 h in which discrete cell detachment was only detect in cultures treated with 100 μg/mL of toxin (Fig 5A).

Cell damage could be attributed to Sat since the cells incubated for 8 h with purified toxin, previously treated with anti-Sat antibodies or PMSF, did not show similar damage (Fig 5B). The presence of the anti-Sat antibodies and PMSF reduced cell detachment by approximately 70 and 90%, respectively (Fig 5C). The results presented are representative of three independent experiments performed in triplicate.

Increasing cell detachment observed over kinetics (Fig 5A) raised interest in assessing cell viability after different contact times with toxin. Due to the presence of *sat* in intestinal bacteria, viability was performed in polarized Caco-2 cells, after 5, 8 and 24 h of contact with 100 μg/mL of Sat.

The viability of adhered and detached cells was evaluated by MTT and Trypan Blue exclusion methods, respectively. The viability of adhered cells was slightly reduced only within 24 h of incubation (Table 3), when there is naturally cell damage due to nutrient consumption from the culture medium and increased metabolites. On the other hand, all detached cells regardless of incubation time, were dead (Table 3).

**Table 3:** Cell viability evaluation of polarized Caco-2 cells after incubation with purified Sat.

	Cell	Methods	Toxin Contact Time		
			5 h	8 h	24 h
**% Cell viability**	Adhered	MTT	95.7%	92%	74.5%
	Detached	Trypan Blue	0%	0%	0%

Considering that alteration in the cytoskeleton must precede cell detachment, the actin filaments from cells incubated for 6 h with Sat were evaluated. After incubation, cells were fixed, permeabilized and stained with DAPI and rhodamine-phalloidin. Sat altered actin filaments from polarized intestinal cells (Fig 5D) in the time preceding the largest cell detachment (Fig 5A).

### Sat internalization in polarized Caco-2 cells

**Fig 6:**
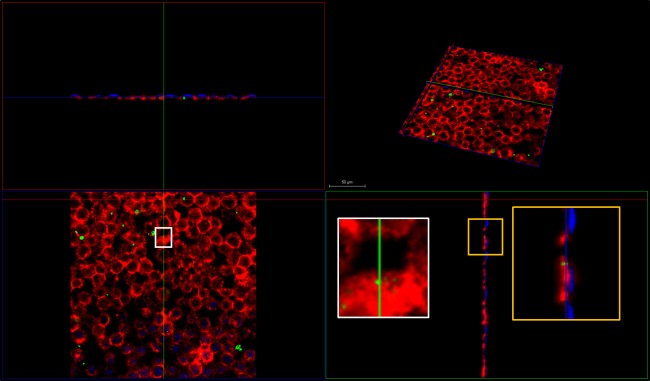
Sat internalization in polarized Caco-2 cells. Cells incubated for 6 h with FITC-labeled Sat (green) were labeled by phalloidin rhodamine (red) and DAPI (blue). The internalization of the Sat was observed by confocal fluorescence microscopy (Leica TCS SP8) and images were obtained via LASX computer software. Bar: 50 μm.

In order to confirm that the observed F-actin change (Fig 5D) was due to toxin presence, Sat internalization in polarized Caco-2 intestinal cells was investigated by confocal laser-scanning microscopy. Cells were incubated with 100 μg/mL FITC-labeled purified Sat for 6 h. After incubation, cells were fixed, permeabilized and stained with DAPI and rhodamine-phalloidin. FITC-Sat fluorescence was detected co-localized with DAPI and rhodamine confirming toxin internalization (Fig 6). Cells incubated with FITC, in the absence of coupled toxin, were used as negative control.

### Effect of Sat toxin on cells of different origins

In addition to the detection of *sat* in UPEC [10], DAEC [21] and EAEC [20], it has also been described as one of the most prevalent SPATE-encoding gene in bacterial strains isolated from neonatal septicemia [23]. The fact that *sat* has been detected in bacteria isolated from different niches, such as the bloodstream and urinary and gastrointestinal tracts aroused the interest to investigate whether cell lines, originating from tissues corresponding to these niches, respond equally to the toxin. To date, there is no report comparing the action of Sat on different cell lines.

**Fig 7:**
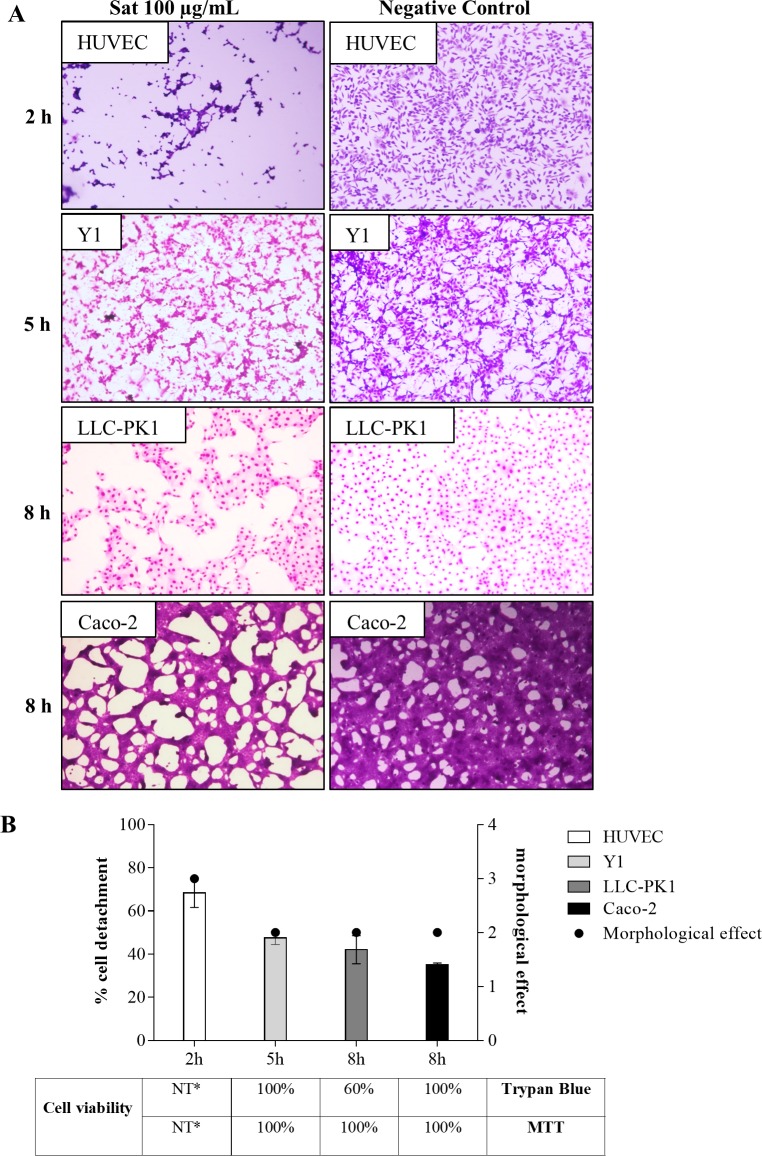
Action of Sat on cell lines originated from different tissues. A) The HUVEC, Y1, LLC-PK1 and Caco-2 cell lines treated with 100 μg/mL of Sat. The minimum time for observation of cell damage in each of the cell lines is shown in the figure. Evaluating of cell damage by light microscopy. Magnifications 100X. B) Summary of the effects of Sat on the different cell lines including, morphological effect, cell detachment and adhered cell viability by MTT and Trypan Blue. NT* Not Tested.

Cell lines LLC-PK1 (kidney-derived), Caco-2 (intestinal-derived) and HUVEC (endothelial-derived) were incubated with 100 μg/mL of Sat during different times. Fig 7A shows the shortest time in which each cell line presented significant cell damage when compared to the control. The HUVEC cells were the most sensitive, since only 2 h of incubation resulted in a cell detachment above 50% with intense morphological alteration (3+) (Fig 7B). Considering cell detachment and incubation time, the Y1 cell line was the second most sensitive followed by the LLC-PK1, while Caco-2 was the less damaged (Fig 7B).

The viability of the cells that remained adherent, post-incubation with the toxin was assessed by the Trypan blue exclusion method and MTT. Only in the LLC-PK1 the reduction of the viability was observed by Trypan blue (60%), which indicates compromised membrane permeability. In the cells of the Y1 and Caco-2 lines, no impairment was detected in Trypan Blue or MTT methods (Fig 7B), although there was cell detachment (Fig 7A). Viability was assessed at the maximum incubation time preceding intense cell detachment, 5 h for Y1 and 8 h for LLC-PK1 and Caco-2 cells (Fig 7B). The high cell detachment of HUVEC observed already within 2 h of contact did not allow the cell viability to be assessed (Fig 7).

The permeability and metabolism of the adherent cells were not altered by contact with Sat. Except for LLC-PK1, 100% of the adhered cells of the other cell lines tested remained viable after incubation with the toxin (Fig 7). The fact that 100% of detached Caco-2 cells were killed due to Sat action (Table 3), the viability of detached HUVEC and LLCP-K1 cells was not investigated.

Our results showed that cell lines tested here representing the urinary and gastrointestinal tracts or endothelium are sensitive to Sat. In all cell lines, some level of cell detachment was observed. However, the sensitivity differed both in the required contact time and in the extent of the observed damage.

## Discussion

Sat is a Class-1 SPATE whose cytopathic effect has been described in kidney and bladder cells [12], since the toxin was originally described in UPEC [10]. Among the diarrheagenic *E*. *coli* pathotypes the presence of *sat* has already been detected in ETEC [16], EPEC [10,17,45], EAEC [10,16,19] and DAEC [16,18,21]. However, among these pathotypes cell damage has only been described in models of DAEC infection. [18,21]. Sat secreted by DAEC was responsible for increasing mucus production in rabbit ileum loops [21] and by the rearrangement of tight junctions associated proteins ZO-1, ZO-3, ocludin and claudin-1 [18]. In EAEC, Sat was detected in the bacterial culture supernatant by mass spectrometry, without any description about its action [19].

SPATEs play an important role in EAEC pathogenesis as they comprise virulence factors involved in cytotoxicity and immunomodulation [5,46]. Among the cytotoxic SPATEs described so far, Pet is undoubtedly the most studied toxin, which is secreted by the EAEC prototype strain 042 [47,48]. Several studies performed with purified Pet led to the determination of its target cells [49] and receptor [50], as well as its intracellular traffic [51]. Although Pet-induced cytotoxicity is associated with the pathogenesis of EAEC [42], the frequency of *pet* in this pathotype does not exceed 20% [19,20,36,52,53], indicating that other SPATEs with equivalent function may be contributing to the cytotoxicity characteristic of EAEC infection. Therefore, Sat may be a candidate for this role, since the prevalence of *sat* is higher than *pet*, being described in approximately 40 to 75% of EAEC strains [19,20,38]. However, as Sat has been identified in UPEC, its function has been better studied in urinary tract infection [10–12] and, to date, there are no reports of its involvement in EAEC infections.

In this work we demonstrated that during the infection of polarized Caco-2 cells with EAEC CV323/77 there was cell detachment, which was inhibited by both anti-Sat and PMSF (Fig 3D). Correlation of Sat with damage in polarized Caco-2 cells has been well demonstrated, but in DAEC infection [18,54]. Sat secreted by one DAEC strain interfered with proteins associated with tight junctions [18,54]. On the other hand, Sat expressed by a probiotic *E*. *coli* does not act as a virulence factor, raising the hypothesis that the action of the same virulence factor may differ according to the bacterial background [22]. The detachment of EAEC-induced polarized Caco-2 cells (Fig 3C and 3D) could reinforce this hypothesis, as this effect has not been described in DAEC-infected polarized Caco-2 cells [18,54].

The secretion of Sat by EAEC strain CV323/77 does not appear to be an exception to this pathotype, since 93.7% of *sat*-positive EAEC strains evaluated in our study secreted Sat in culture supernatants (Fig 4). Studies have shown that strains of EAEC often harbor two members of the SPATE family, generally *pic* and one cytotoxic SPATE [7]. The EAEC CV323/77 studied here harbors *pic* and *sat* (Table 1). Pic is an immunomodulatory SPATE with no cytotoxic activity [14]. As the relevance of Pic in EAEC pathogenesis is its role in colonization, we can consider that, in the presence of *sat* and *pic*, as in EAEC CV323/77 (Table 1), two aspects of pathogenesis would be present, *i*.*e*. gut colonization and cytotoxicity.

The identity of Sat present in the culture supernatant of DEC/Sat+ strain was confirmed by MS/LC/MS mass spectrometry. Alignment of Sat nucleotide sequences, of EAEC CV323/77 and DEC/Sat+, with *sat* of UPEC strain CFT073 (GenBank accession number: AF289092.1) identified two point mutations (GenBank accession numbers: LT855558 and LT855559). When compared to the reference strain (Table 2), in both strains of this study the exchange were valine for alanine, both of the same group (position 669), and threonine for alanine (position 771), a polar amino acid substituted by an apolar. The fact that mutations occurred outside of the serine protease catalytic triad (histidine, aspartic acid and serine) do not assure the functionality of the protein. Mutation of amino acid residues at the non-active site may contribute to important conformational change [55]. However, regardless of the two mutations identified (Table 2), the toxin secreted by DEC/Sat+ showed activity on cultivated cells (Fig 5). Interestingly, one of the mutations observed in the purified Sat (position 669) was also described in the Sat secreted by the probiotic strain Nissle 1917 [22].

We demonstrated that Sat enters into polarized intestinal cells (Fig 6) and induces cell detachment in a time dependent manner (Fig 5A), interfering with actin filaments (Fig 5D).

The presence of Sat in UPEC supported further studies using kidney and bladder cells, which could allow to study the toxin’s mechanism of action and its possible cellular targets [10–12]. Similarly, the description of Sat in DAEC conducted to studies using polarized intestinal cells, which described increased paracellular permeability that could contribute to bacterial translocation [18,22,54]. Currently, our group and others have observed high prevalence of *sat* in strains isolated from bacteremia [23]. Moreover, *sat* was also identified in an isolated strain of fatal meningitis [24].

The finding of *sat* in strains isolated from different sites of infection raises the idea that Sat may have some relevance in addition to its role in the urinary and gastrointestinal infections [56]. To investigate this possibility, Sat was employed in a comparative study of the toxin action on cells from the urinary tract (Y1 and LLC-PK1), gastrointestinal tract (Caco-2) and endothelium (HUVEC), as representatives of different niches in which *sat*^*+*^-positive *E*. *coli* strains have been identified. Our results showed that the toxin activity differs in terms of induced damage and the required contact time (Fig 7). The action of Sat was more prominent in HUVEC cells. The morphological effect that was similar between urinary and gastrointestinal tracts derived cells was also higher in the endothelial cells incubated for a shorter time (Fig 7B).

Although present, the detachment of Caco-2 cells was the lowest when compared to the other lines, since the observed damage was more like "cell shrinkage" than detachment *per se* (Fig 7A). Sat in colorectal adenocarcinoma Caco-2 cells induces redistribution of the ZO1, ZO3 and occludin proteins leading to an increase in intercellular permeability [18]. Interestingly, the detachment of Caco-2 cells infected by the pathogenic strains that secrete Sat was higher (Fig 3C and 3D) than the observed in Caco-2 cells incubated with the purified toxin (Fig 7B). Pathogenic bacteria can carry other virulence factors that could enhance the damage during infection. In both cases, in infection and in incubation with purified Sat, PMSF and anti-Sat antibody significantly inhibited cell detachment confirming that the observed damage was due to Sat (Figs 3A and 5C). On the other hand, the cell detachment observed in Y1 and LLC-PK1 lines was similar (about 50% cell detachments) (Fig 7B). Even so, Y1 cells were more sensitive to Sat as the same cell detachment was observed with shorter incubation time (Fig 7B). In kidney and bladder cells, Sat interacts with fodrin, which is associated with F-actin [11]. This could explain the cell detachment observed in cell lines derived from the urinary tract.

Here we demonstrate that Sat enters polarized Caco-2 cells (Fig 6), interferes with F-actin (Fig 5D), which probably impairs focal adhesion, favoring cell detachment (Fig 5B). However, polarization of Caco-2 cells may be an important aspect in reducing cell detachment when compared to other cell lines (Fig 7).

Moreover, our results showed that HUVEC cells are the most sensitive cell line to Sat (Fig 7). Increased sensitivity of endothelial cells to Sat and the high prevalence of *sat* in bacterial strains isolated from blood may have a relevant role in the pathogenesis of sepsis. Bacteria isolated from bacteremia frequently reach the bloodstream as a result of a urinary tract infection or intestinal translocation. Thus, a particular virulence factor could play a distinct role depending on the niche of the infection. Here, we suggest that Sat may also play a role in niches other than the intestinal and urinary tracts. However, once the bacterium reaches the bloodstream, the importance of the toxin in the pathogenesis seems to be exacerbated. Studies on the mechanism of action of Sat in endothelial cells and with bacteria isolated from septicemia are being performed by our group.

## Supporting information

S1 Fig Raw image for the production of Fig 1. (TIF)

S2 Fig Raw image for the production of Fig 2B. (TIF)

S3 Fig Raw image for the production of Fig 2C. (TIF)

S4 Fig Raw image for the production of Fig 3B. (TIF)

S5 Fig Raw image for the production of Fig 3E. (TIF)

## References

[1] Rao MB, Tanksale AM, Ghatge MS, Deshpande V V. Molecular and Biotechnological Aspects of Microbial Proteases. Microbiol Mol Biol Rev. 1998;62: 597–635. papers2://publication/uuid/E58ABF6D-8C97-4209-810D-A452EE30B2CD 9729602 10.1128/mmbr.62.3.597-635.1998 PMC98927

[2] Frees D, Brøndsted L, Ingmer H. Bacterial Proteases and Virulence. Regulated Proteolysis in Microorganisms. 2013 pp. 195–222. 10.1007/978-94-007-5940-4

[3] Page MJ, Di Cera E. Serine peptidases: Classification, structure and function. Cellular and Molecular Life Sciences. 2008 pp. 1220–1236. 10.1007/s00018-008-7565-9 18259688 PMC11131664

[4] Henderson IR, Navarro-Garcia F, Nataro JP. The great escape: Structure and function of the autotransporter proteins. Trends in Microbiology. 1998 pp. 370–378. 10.1016/s0966-842x(98)01318-3 9778731

[5] Henderson IR, Navarro-Garcia F, Desvaux M, Fernandez RC, Ala’Aldeen D. Type V Protein Secretion Pathway: the Autotransporter Story Type V Protein Secretion Pathway: the Autotransporter Story. Microbiol Mol Biol Rev. 2004;68: 692–744. 10.1128/MMBR.68.4.692-744.2004 15590781 PMC539010

[6] Dautin N. Serine protease autotransporters of Enterobacteriaceae (SPATEs): Biogenesis and function. Toxins. 2010 pp. 1179–1206. 10.3390/toxins2061179 22069633 PMC3153244

[7] Ruiz-Perez F, Nataro JP. Bacterial serine proteases secreted by the autotransporter pathway: Classification, specificity, and role in virulence. Cell Mol Life Sci. 2014;71: 745–770. 10.1007/s00018-013-1355-8 23689588 PMC3871983

[8] Grijpstra J, Arenas J, Rutten L, Tommassen J. Autotransporter secretion: Varying on a theme. Res Microbiol. 2013;164: 562–582. 10.1016/j.resmic.2013.03.010 23567321

[9] Klauser T, Pohlner J, Meyer TF. The secretion pathway of IgA protease‐type proteins in gram‐negative bacteria. BioEssays. 1993 pp. 799–805. 10.1002/bies.950151205 8141798

[10] Guyer DM, Henderson IR, Nataro JP, Mobley HLT. Identification of Sat, an autotransporter toxin produced by uropathogenic *Escherichia coli*. Mol Microbiol. 2000;38: 53–66. 10.1046/j.1365-2958.2000.02110.x 11029690

[11] Maroncle NM, Sivick KE, Brady R, Stokes FE, Mobley HLT. Protease activity, secretion, cell entry, cytotoxicity, and cellular targets of secreted autotransporter toxin of uropathogenic *Escherichia coli*. Infect Immun. 2006;74: 6124–6134. 10.1128/IAI.01086-06 16954394 PMC1695523

[12] Guyer DM, Radulovic S, Jones FE, Mobley HLT. Sat, the secreted autotransporter toxin of uropathogenic *Escherichia coli*, is a vacuolating cytotoxin for bladder and kidney epithelial cells. Infect Immun. 2002;70: 4539–4546. 10.1128/IAI.70.8.4539-4546.2002 12117966 PMC128167

[13] Liévin-Le Moal V, Comenge Y, Ruby V, Amsellem R, Nicolas V, Servin AL. Secreted autotransporter toxin (Sat) triggers autophagy in epithelial cells that relies on cell detachment. Cell Microbiol. 2011;13: 992–1013. 10.1111/j.1462-5822.2011.01595.x 21501364

[14] Dutta PR, Dutta PR, Cappello R, Cappello R, Navarro-garc F, Navarro-garc F, et al Functional Comparison of Serine Protease Autotransporters of. Society. 2002;70: 7105–7113. 10.1128/IAI.70.12.7105 PMC133081 12438392

[15] Niyogi SK, Vargas M, Villa J. Prevalence of the sat, set and sen genes among diverse serotypes of *Shigella flexneri* strains isolated from patients with acute diarrhoea. Clin Microbiol Infect. European Society of Clinical Infectious Diseases; 2004;10: 574–576. 10.1111/j.1469-0691.2004.00897.x 15191388

[16] Taddei CR, Moreno ACR, Fernandes Filho A, Montemor LPG, Martinez MB. Prevalence of secreted autotransporter toxin gene among diffusely adhering *Escherichia coli* isolated from stools of children. FEMS Microbiol Lett. 2003;227: 249–253. 10.1016/S0378-1097(03)00688-8 14592716

[17] Abreu AG, Bueris V, Porangaba TM, Sircili MP, Navarro-Garcia F, Elias WP. Autotransporter protein-encoding genes of diarrheagenic *Escherichia coli* are found in both typical and atypical enteropathogenic *E*. *coli* strains. Appl Environ Microbiol. 2013;79: 411–414. 10.1128/AEM.02635-12 23104414 PMC3536084

[18] Guignot J, Chaplais C, Coconnier-Polter MH, Servin AL. The secreted autotransporter toxin, Sat, functions as a virulence factor in Afa/Dr diffusely adhering *Escherichia coli* by promoting lesions in tight junction of polarized epithelial cells. Cell Microbiol. 2007;9: 204–221. 10.1111/j.1462-5822.2006.00782.x 16903846

[19] Boisen N, Ruiz-Perez F, Scheutz F, Krogfelt KA, Nataro JP. Short Report: High Prevalence of Serine Protease Autotransporter Cytotoxins among Strains of Enteroaggregative *Escherichia coli*. Am J Trop Med Hyg. 2009;80: 294–301. 19190229 PMC2660206

[20] Andrade FB, Abreu AG, Nunes KO, Gomes TAT, Piazza RMF, Elias WP. Distribution of serine protease autotransporters of Enterobacteriaceae in typical and atypical enteroaggregative *Escherichia coli*. Infect Genet Evol. Elsevier B.V.; 2017;50: 83–86. 10.1016/j.meegid.2017.02.018 28254427

[21] Taddei CR, Fasano A, Ferreira AJP, Trabulsi LR, Martinez MB. Secreted autotransporter toxin produced by a diffusely adhering *Escherichia coli* strain causes intestinal damage in animal model assays. FEMS Microbiol Lett. 2005;250: 263–269. 10.1016/j.femsle.2005.07.013 16098687

[22] Toloza L, Giménez R, Fábrega MJ, Alvarez CS, Aguilera L, Cañas MA, et al The secreted autotransporter toxin (Sat) does not act as a virulence factor in the probiotic *Escherichia coli* strain Nissle 1917. BMC Microbiol. BMC Microbiology; 2015;15: 250 10.1186/s12866-015-0591-5 26518156 PMC4628265

[23] Tapader R, Chatterjee S, Singh AK, Dayma P, Haldar S, Pal A, et al The high prevalence of serine protease autotransporters of Enterobacteriaceae (SPATEs) in *Escherichia coli* causing neonatal septicemia. Eur J Clin Microbiol Infect Dis. 2014;33: 2015–2024. 10.1007/s10096-014-2161-4 24924922

[24] Iqbal J, Dufendach KR, Wellons JC, Kuba MG, Nickols HH, Gómez-Duarte OG, et al Lethal neonatal meningoencephalitis caused by multi-drug resistant, highly virulent Escherichia coli. Infect Dis (Auckl). 2016;48: 461–466. 10.3109/23744235.2016.1144142 27030919 PMC4818964

[25] Miajlovic H, Mac Aogáin M, Collins CJ, Rogers TR, Smith SG. Characterization of *Escherichia coli* bloodstream isolates associated with mortality. J Med Microbiol. 2015; 71–79. 10.1099/jmm.0.000200 26518234

[26] Mahjoub-Messai F, Bidet P, Caro V, Diancourt L, Biran V, Aujard Y, et al *Escherichia coli* isolates causing bacteremia via gut translocation and urinary tract infection in young infants exhibit different virulence genotypes. J Infect Dis. 2011;203: 1844–1849. 10.1093/infdis/jir189 21550999

[27] Clermont O, Couffignal C, Blanco J, Mentré F, Picard B, Denamur E. Two levels of specialization in bacteraemic *Escherichia coli* strains revealed by their comparison with commensal strains. Epidemiol Infect. 2017;145: 872–882. 10.1017/S0950268816003010 28029088 PMC9507814

[28] Nojoomi F, Ghasemian A. The relation of phylogroups, serogroups, virulence factors and resistance pattern of *Escherichia coli* isolated from children with septicemia. New Microbes New Infect. 2019; 10.1016/j.nmni.2019.100517 31080621 PMC6501060

[29] Merino I, Porter SB, Johnston BD, Clabots C, Shaw E, Horcajada JP, et al Virulence genes and subclone status as markers of experimental virulence in a murine sepsis model among *Escherichia coli* sequence type 131 clinical isolates from Spain. PLoS One. 2017; 10.1371/journal.pone.0188838 29190804 PMC5708792

[30] Campos LC, Franzolin MR, Trabulsi LR. Diarrheagenic *Escherichia coli* categories among the traditional enteropathogenic *E*. *coli* O serogroups—A review. Memorias do Instituto Oswaldo Cruz. 2004 pp. 545–552. 10.1590/s0074-02762004000600001 15558161

[31] Freitas Do Valle GR, Tardelli Gomes TA, Irino K, Trabulsi LR. The traditional enteropathogenic Escherichia coli (EPEC) serogroup O125 comprises serotypes which are mainly associated with the category of enteroaggregative *E*. *coli*. FEMS Microbiol Lett. 1997;152: 95–100. 10.1111/j.1574-6968.1997.tb10414.x 9228775

[32] Nataro JP, Steiner T, Guerrant RL. Enteroaggregative *Escherichia coli*. Emerg Infect Dis. 1998;4: 251–261. 10.3201/eid0402.980212 9621195 PMC2640136

[33] Sambrook J, Fritsch EF, Maniatis T. Molecular cloning. Society. 1989;68: 1232–1239. 10.1128/AEM.68.3.1232

[34] Baker RD, Baker SS, Larosa K. Polarized Caco-2 cells—Effect of reactive oxygen metabolites on enterocyte barrier function. Dig Dis Sci. 1995; 10.1007/BF02064358 7895534

[35] Cravioto A, Gross RJ, Scotland SM, Rowe B. An adhesive factor found in strains of *Escherichia coli* belonging to the traditional infantile enteropathogenic serotypes. Curr Microbiol. 1979;3: 95–99. 10.1007/BF02602439

[36] Elias WP, Uber AP, Tomita SK, Trabulsi LR, Gomes TAT. Combinations of putative virulence markers in typical and variant enteroaggregative *Escherichia coli* strains from children with and without diarrhoea. Epidemiol Infect. 2002;129: 49–55. 10.1017/s0950268802007136 12211596 PMC2869874

[37] Schmidt H, Schmidt H, Hemmrich U, Hemmrich U, Jelacic S, Jelacic S, et al Identification and Characterization of a Novel Genomic Island Integrated at. Society. 2001;69: 6863–6873. 10.1128/IAI.69.11.6863 PMC100065 11598060

[38] Boisen N, Scheutz F, Rasko DA, Redman JC, Persson S, Simon J, et al Genomic characterization of enteroaggregative *Escherichia coli* from children in Mali. J Infect Dis. 2012;205: 431–444. 10.1093/infdis/jir757 22184729 PMC3256949

[39] Andrade FB, Gomes TAT, Elias WP. A sensitive and specific molecular tool for detection of both typical and atypical enteroaggregative *Escherichia coli*. J Microbiol Methods. 2014;106: 16–18. 10.1016/j.mimet.2014.07.030 25108292

[40] Oh KH, Kim DW, Jung SM, Cho SH. Molecular characterization of enterotoxigenic *Escherichia coli* strains isolated from diarrheal patients in Korea during 2003–2011. PLoS One. 2014; 10.1371/journal.pone.0096896 24841334 PMC4026316

[41] Saidi RF, Sears CL. *Bacteroides fragilis* Toxin Rapidly Intoxicates Human Intestinal Epithelial Cells (HT29 / C 1) In Vitro. Infect Immun. 1996;64: 5029–34. 8945542 10.1128/iai.64.12.5029-5034.1996 PMC174484

[42] Navarro-García F, Sears C, Eslava C, Cravioto A, Nataro JP. Cytoskeletal effects induced by Pet, the serine protease enterotoxin of enteroaggregative *Escherichia coli*. Infect Immun. 1999;67: 2184–2192. 10225873 10.1128/iai.67.5.2184-2192.1999 PMC115956

[43] Kyte J, Doolittle RF. A simple method for displaying the hydropathic character of a protein. J Mol Biol. 1982; 10.1016/0022-2836(82)90515-0 7108955

[44] Fraga S. Theoretical prediction of protein antigenic determinants from amino acid sequences. Can J Chem. NRC Research Press; 1982;60: 2606–2610.

[45] Souza Marina B; Taddei Carla R; Mukai Lilian; Gilio, Alfredo E; Racz Maria L; Silva Luzinete; Ejzenberg Bernardo; Okay Yassuhiko. ECM. Perfil etiológico da diarréias agudas de crianças atendidas em Säo Paulo / Etiologic prosfile of acute diarrhea in children in the city of Säo Paulo. J pediatr (Rio J). 2002;78: 34–38. 10.1590/S0021-75572002000100008 14647809

[46] Navarro-Garcia F, Elias WP. Autotransporters and virulence of enteroaggregative *E*. *coli*. Gut Microbes. 2011;2 10.4161/gmic.2.1.14933 21637014

[47] Eslava C, Navarro-García F, Czeczulin JR, Henderson IR, Cravioto A, Nataro JP. Pet, an autotransporter enterotoxin from enteroaggregative *Escherichia coli*. Infect Immun. 1998;66: 3155–3163. 9632580 10.1128/iai.66.7.3155-3163.1998 PMC108327

[48] Navarro-Garcia F. Enteroaggregative *Escherichia coli* plasmid-encoded toxin. Futur Microbiol. 2010;5: 1005–1013. 10.2217/fmb.10.69 20632801

[49] Villaseca JM, Navarro-Garcia F, Mendoza-Hernandez G, Nataro JP, Cravioto A, Eslava C. Pet toxin from enteroaggregative *Escherichia coli* produces cellular damage associated with fodrin disruption. Infect Immun. 2000;68: 5920–5927. 10.1128/iai.68.10.5920-5927.2000 10992503 PMC101555

[50] Nava-Acosta R, Navarro-Garcia F. Cytokeratin 8 is an epithelial cell receptor for pet, a cytotoxic serine protease autotransporter of Enterobacteriaceae. MBio. 2013;4 10.1128/mBio.00838-13 24327340 PMC3870265

[51] Navarro-García F, Canizalez-Roman A, Burlingame KE, Teter K, Vidal JE. Pet, a non-AB toxin, is transported and translocated into epithelial cells by a retrograde trafficking pathway. Infect Immun. 2007;75: 2101–2109. 10.1128/IAI.01515-06 17296748 PMC1865752

[52] Czeczulin JR, Whittam TS, Henderson IR, Navarro-Garcia F, Nataro JP. Phylogenetic analysis of enteroaggregative and diffusely adherent *Escherichia coli*. Infect Immun. 1999;67: 2692–2699. 10338471 10.1128/iai.67.6.2692-2699.1999 PMC96572

[53] Piva IC, Pereira AL, Ferraz LR, Silva RSN, Vieira AC, Blanco JJE, et al Virulence markers of enteroaggregative *Escherichia coli* isolated from children and adults with diarrhea in Brasília, Brazil. J Clin Microbiol. 2003;41: 1827–1832. 10.1128/JCM.41.5.1827-1832.2003 12734212 PMC154701

[54] Nicolas V, Moal VL Le. Antisecretory factor peptide AF-16 inhibits the secreted autotransporter toxin-stimulated transcellular and paracellular passages of fluid in cultured human enterocyte-like cells. Infect Immun. 2015; 10.1128/IAI.02759-14 25534938 PMC4333461

[55] Ali A, Azam MW, Khan AU. Non-active site mutation (Q123A) in New Delhi metallo-β-lactamase (NDM-1) enhanced its enzyme activity. Int J Biol Macromol. 2018; 10.1016/j.ijbiomac.2018.02.091 29454953

[56] Welch RA. Uropathogenic *Escherichia coli*-Associated Exotoxins. Microbiol Spectr. 2016;4: 35–41. 10.1128/microbiolspec.UTI-0011-2012 27337488 PMC4920080

[57] Henderson IR, Czeczulin J, Eslava C, Noriega F, Nataro JP. Characterization of Pic, a secreted protease of *Shigella flexneri* and enteroaggregative *Escherichia coli*. Infect Immun. 1999;67: 5587–5596. 10531204 10.1128/iai.67.11.5587-5596.1999 PMC96930

[58] Torres AG, Perna NT, Burland V, Ruknudin A, Blattner FR, Kaper JB. Characterization of Cah, a calcium-binding and heat-extractable autotransporter protein of enterohaemorrhagic *Escherichia coli*. Mol Microbiol. 2002;45: 951–966. 10.1046/j.1365-2958.2002.03094.x 12180916

[59] Wells TJ, Sherlock O, Rivas L, Mahajan A, Beatson SA, Torpdahl M, et al EhaA is a novel autotransporter protein of enterohemorrhagic *Escherichia coli* O157:H7 that contributes to adhesion and biofilm formation. Environ Microbiol. 2008;10: 589–604. 10.1111/j.1462-2920.2007.01479.x 18237301

[60] Easton DM, Totsika M, Allsopp LP, Phan MD, Idris A, Wurpel DJ, et al Characterization of EhaJ, a new autotransporter protein from enterohemorrhagic and enteropathogenic *Escherichia coli*. Front Microbiol. 2011;2 10.3389/fmicb.2011.00002 21687429 PMC3108271

