# Supplementary figures and images for: Secreted autotransporter toxin (Sat) induces cell damage during enteroaggregative Escherichia coli infection
Source: PLoS One. 2020 Feb 21;15(2):e0228959. doi: 10.1371/journal.pone.0228959 (PMC7034920; doi:10.1371/journal.pone.0228959)

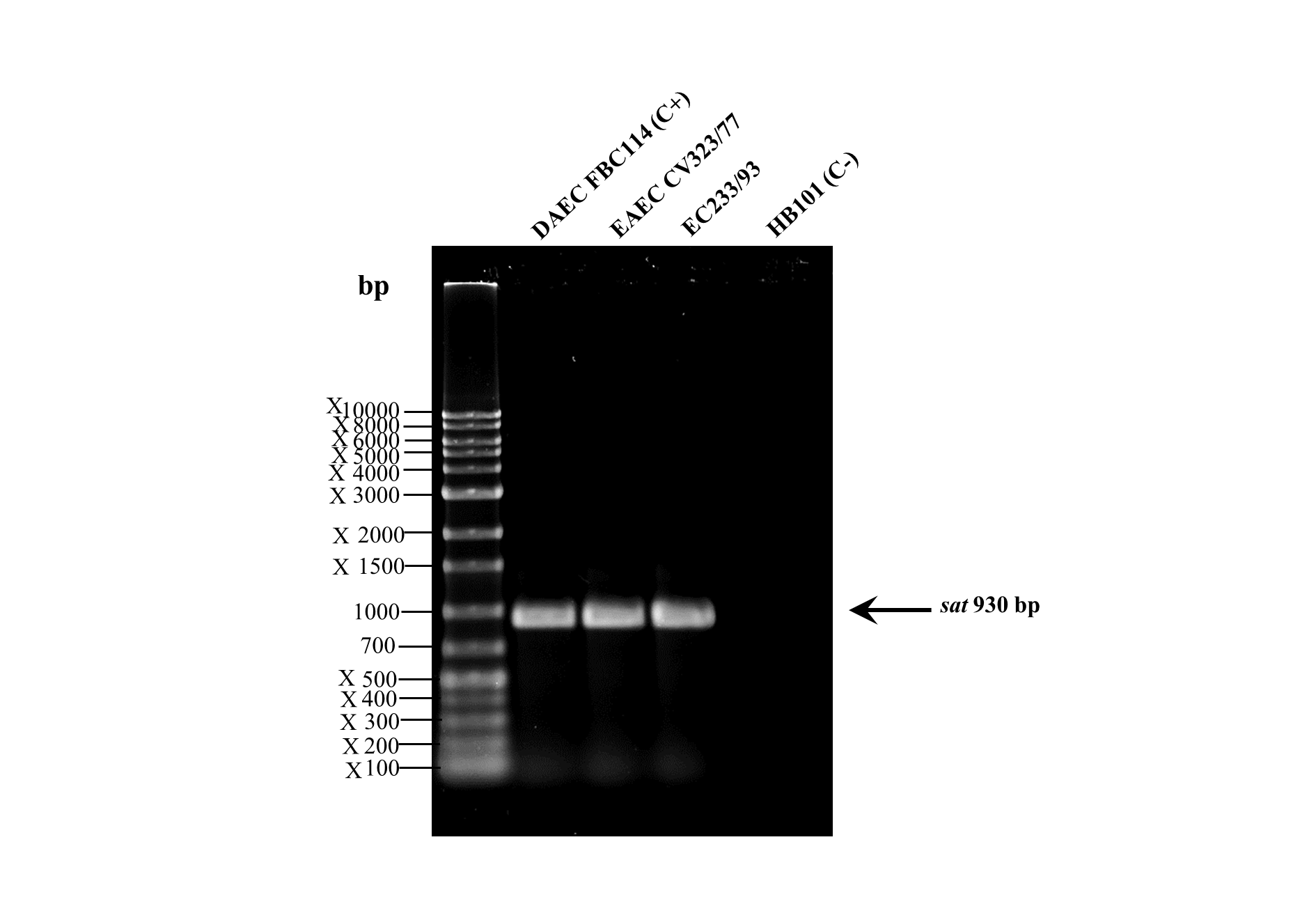

Supplement: S1 Fig — (TIF) [file pone.0228959.s001.tif]

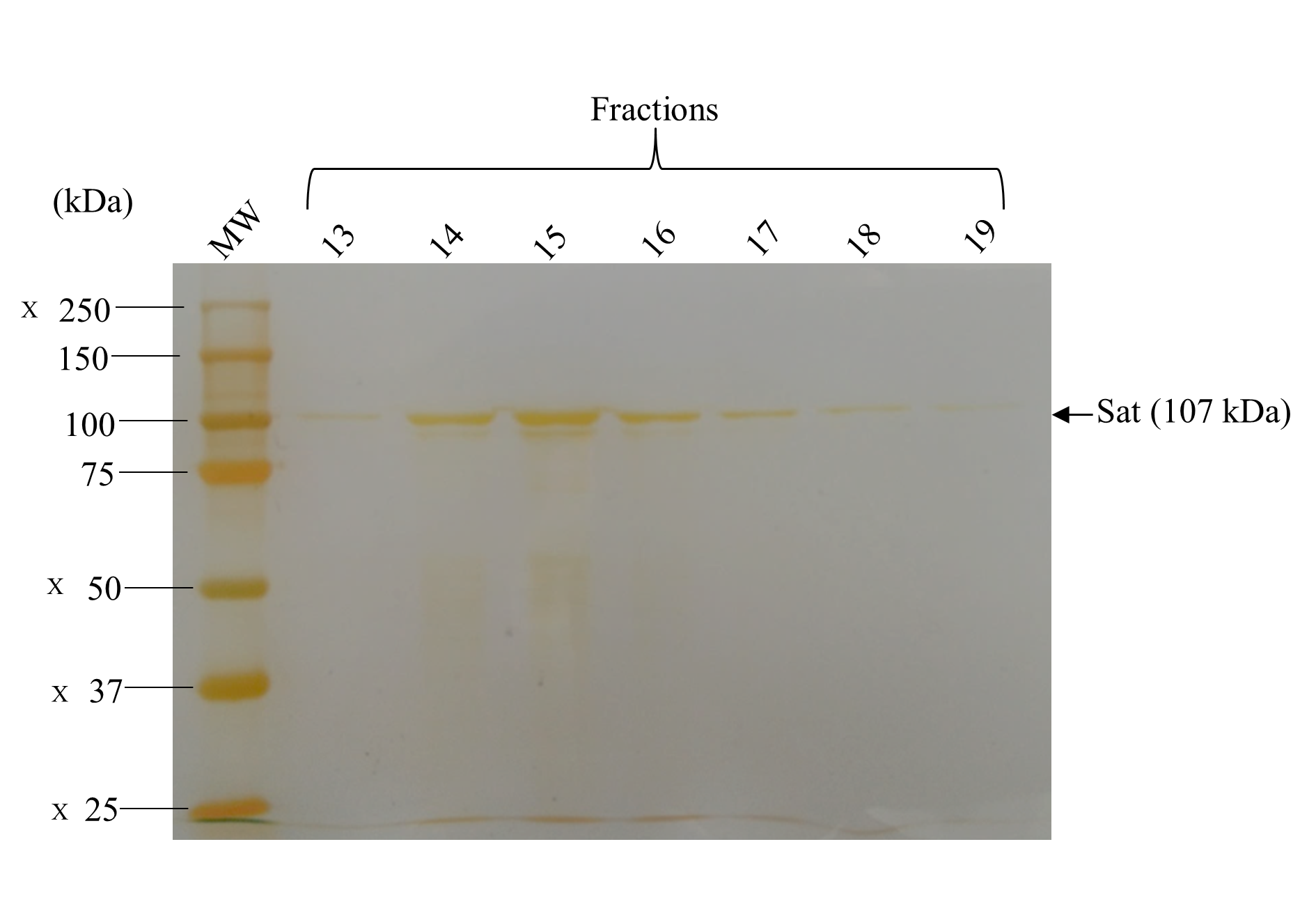

Supplement: S2 Fig — (TIF) [file pone.0228959.s002.tif]

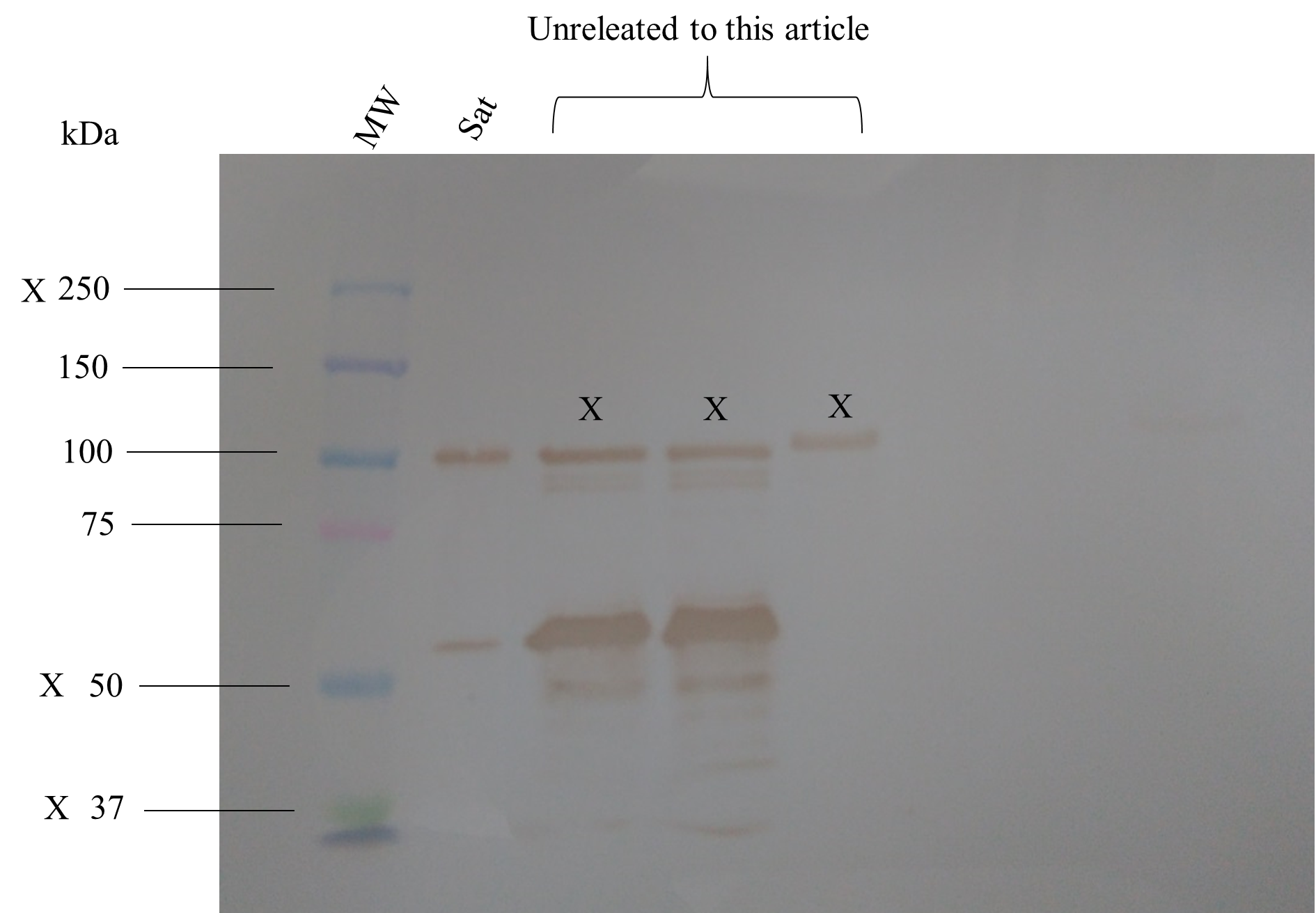

Supplement: S3 Fig — (TIF) [file pone.0228959.s003.tif]

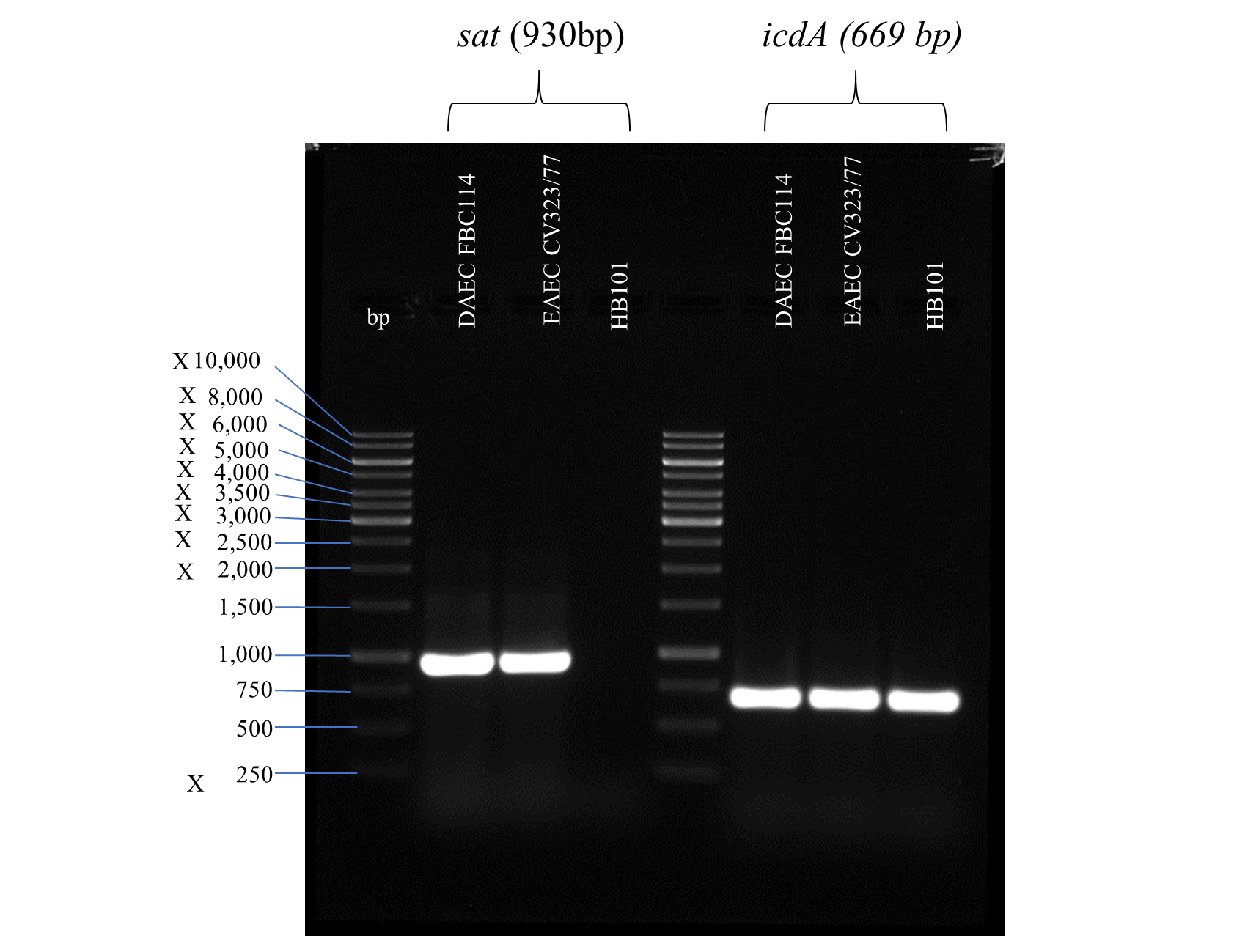

Supplement: S4 Fig — (TIF) [file pone.0228959.s004.tif]

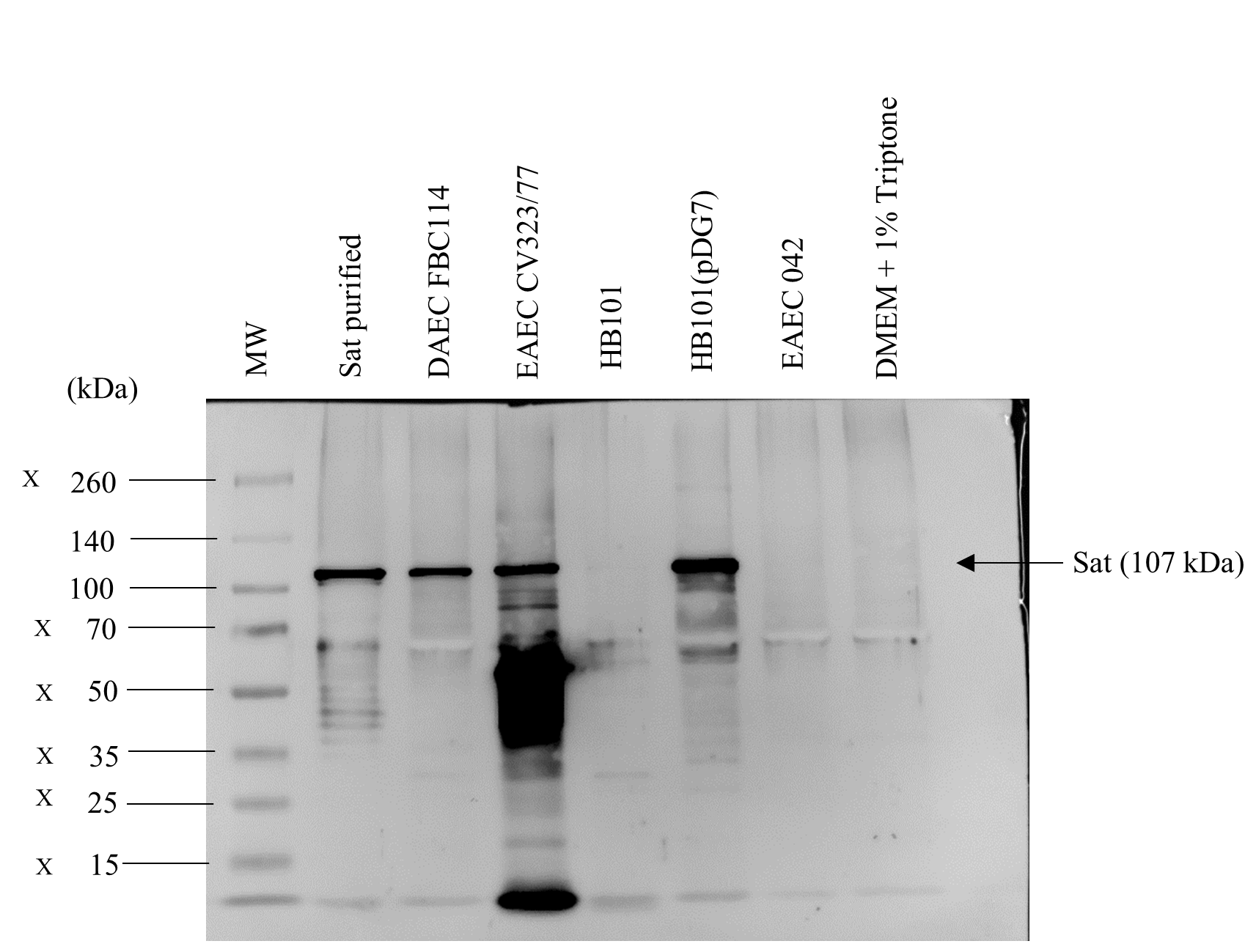

Supplement: S5 Fig — (TIF) [file pone.0228959.s005.tif]
